# Supplementary material for: ALK Inhibitors-Induced M Phase Delay Contributes to the Suppression of Cell Proliferation
Source: Cancers (Basel). 2020 Apr 24;12(4):1054. doi: 10.3390/cancers12041054 (PMC7226408; doi:10.3390/cancers12041054)
Supplement: Supplementary file 1 [file cancers-12-01054-s001.pdf]

cancers

MDPI

Article

ALK inhibitors-induced M phase delay contributes to the suppression of cell proliferation

Sirajam Munira, Ryuzaburo Yuki, Youhei Saito and Yuji Nakayama

Supplementary Materials

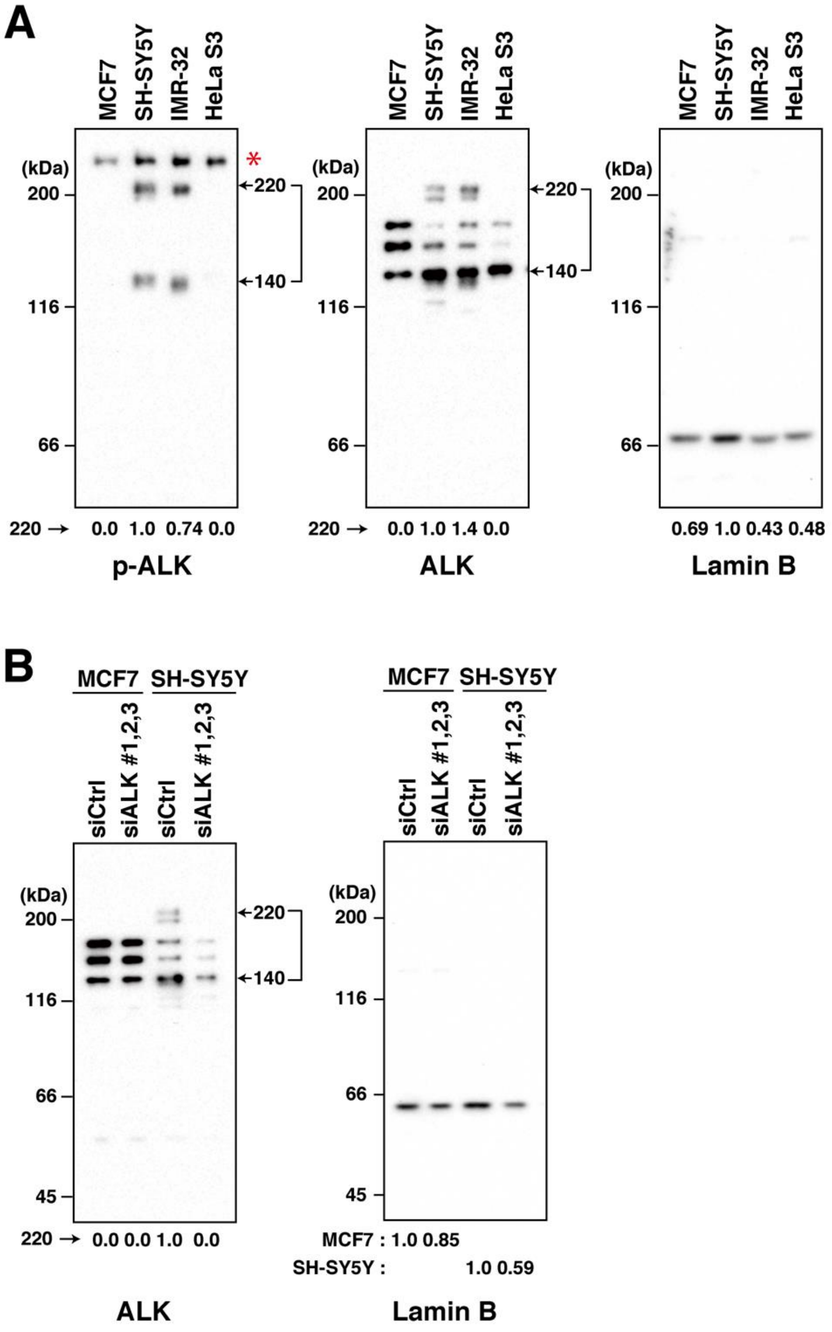

Figure S1. Detection of ALK protein expression in ALK-positive and negative cells. **(A)** Cell lysates were prepared from indicated cells and examined by Western blotting with anti-pALK-Y1507, anti-ALK, and anti-lamin B (loading control) antibodies. Asterisk indicates non-specific bands. **(B)** MCF7

and SH-SY5Y cells were transfected with total 60 pmol of three ALK-targeting siRNAs (siALK #1-3) or nontargeting siRNA (siCtrl). At 70 h after transfection, whole cell lysates were prepared and analyzed by Western blotting with anti-ALK and anti-lamin B (loading control) antibodies. The band intensities were measured and their ratios to that in SH-SY5Y (upper panel) or siCtrl-treated cells (lower panel) are shown below the blots.

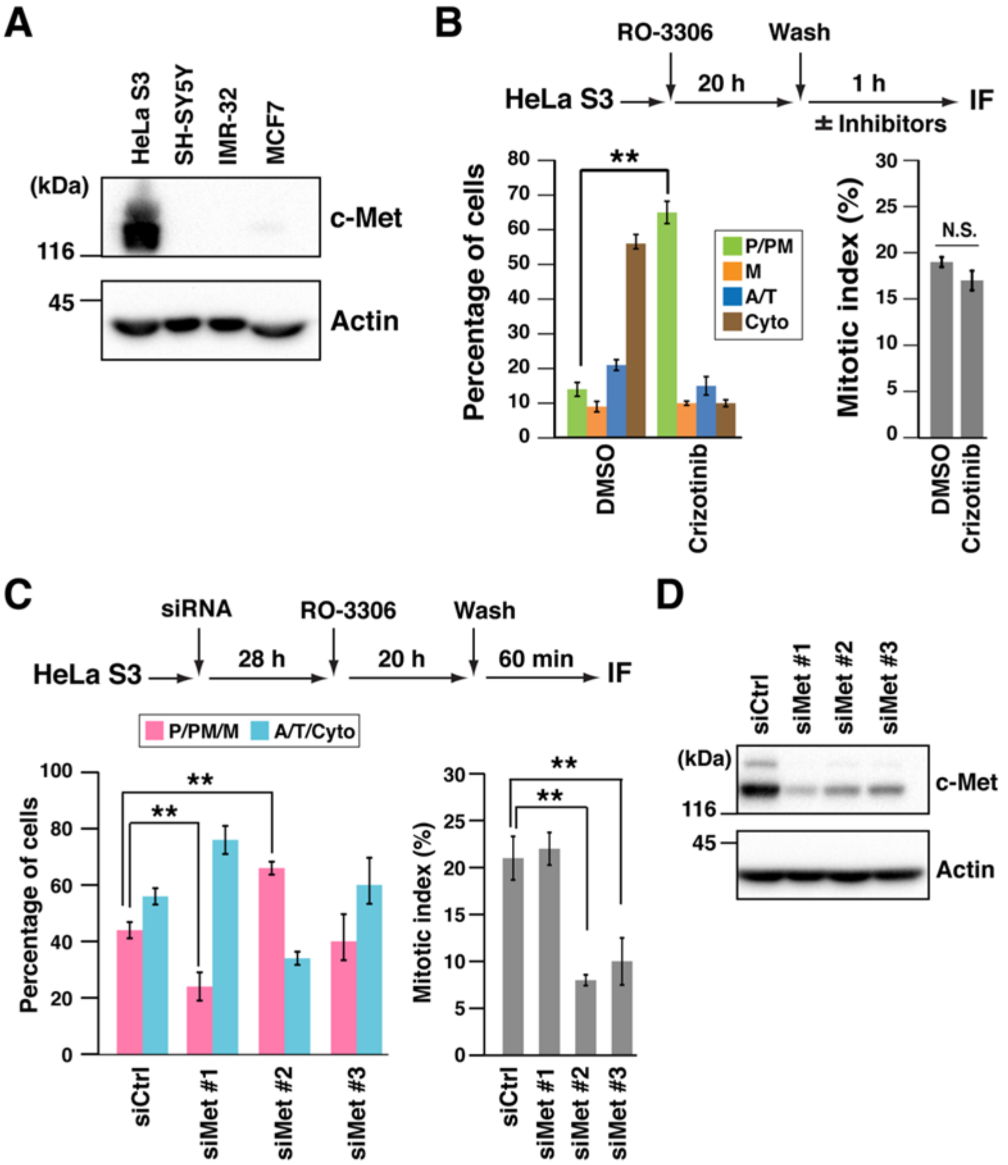

Figure S2. Effect of c-Met inhibition on M-phase progression. **(A)** Whole cell lysates were examined by Western blotting with anti-Met and anti-actin (loading control) antibodies. **(B)** HeLa S3 cells were synchronized with 6 μM RO-3306. After release from RO-3306, HeLa S3 cells were incubated with 1 μM crizotinib for 1 h. As described in Figure 1C, cells were divided into four groups. The percentages of cells of each group ($n > 253$ in each experiment) and mitotic indices (n > 476 in each experiment) are plotted as the mean ± SD calculated from three independent experiments. HeLa S3 cells were treated with c-Met-targeting siRNA and nontargeting siCtrl. **(C)** At 28 h after transfection, cells were synchronized with RO-3306 and released in fresh media for 60 min. M phase cells were classified into two groups: before (P/PM/M) and after (A/T/Cyto) anaphase onset. Percentages of cells (n > 247 in each experiment) and mitotic indices ($n > 507$ in each experiment) are plotted as the mean ± SD calculated from three independent experiments. Student's t-test and the Tukey–Kramer multiple comparison test were used to calculate p values. $^{**}p < 0.01$, N.S., not significant. **(D)** After 48 h of transfection, whole cell lysates were examined by Western blotting with indicated antibodies.

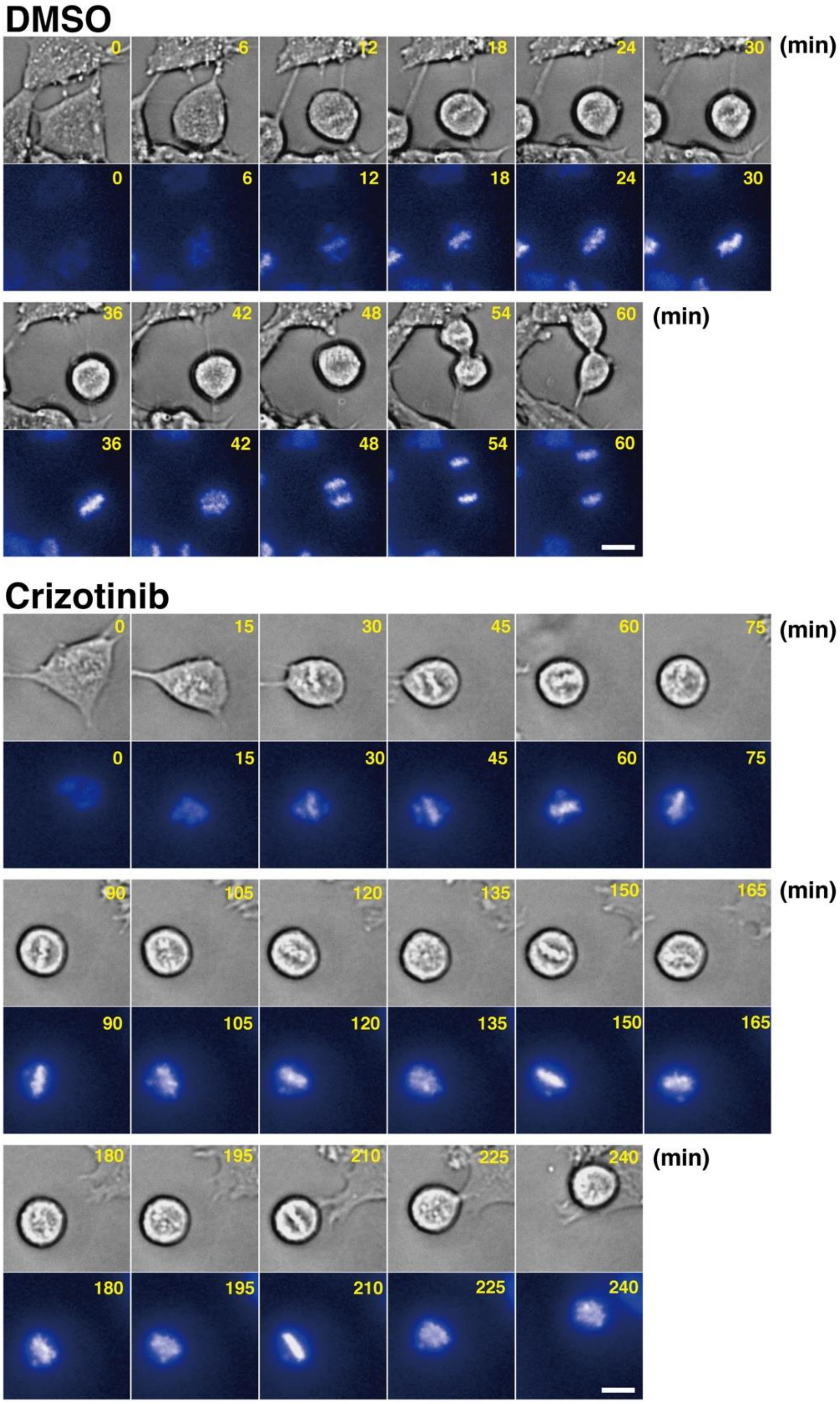

Figure S3. Time-lapse images of Figure 2 in short interval of time are shown. Scale bar, 10 μm.

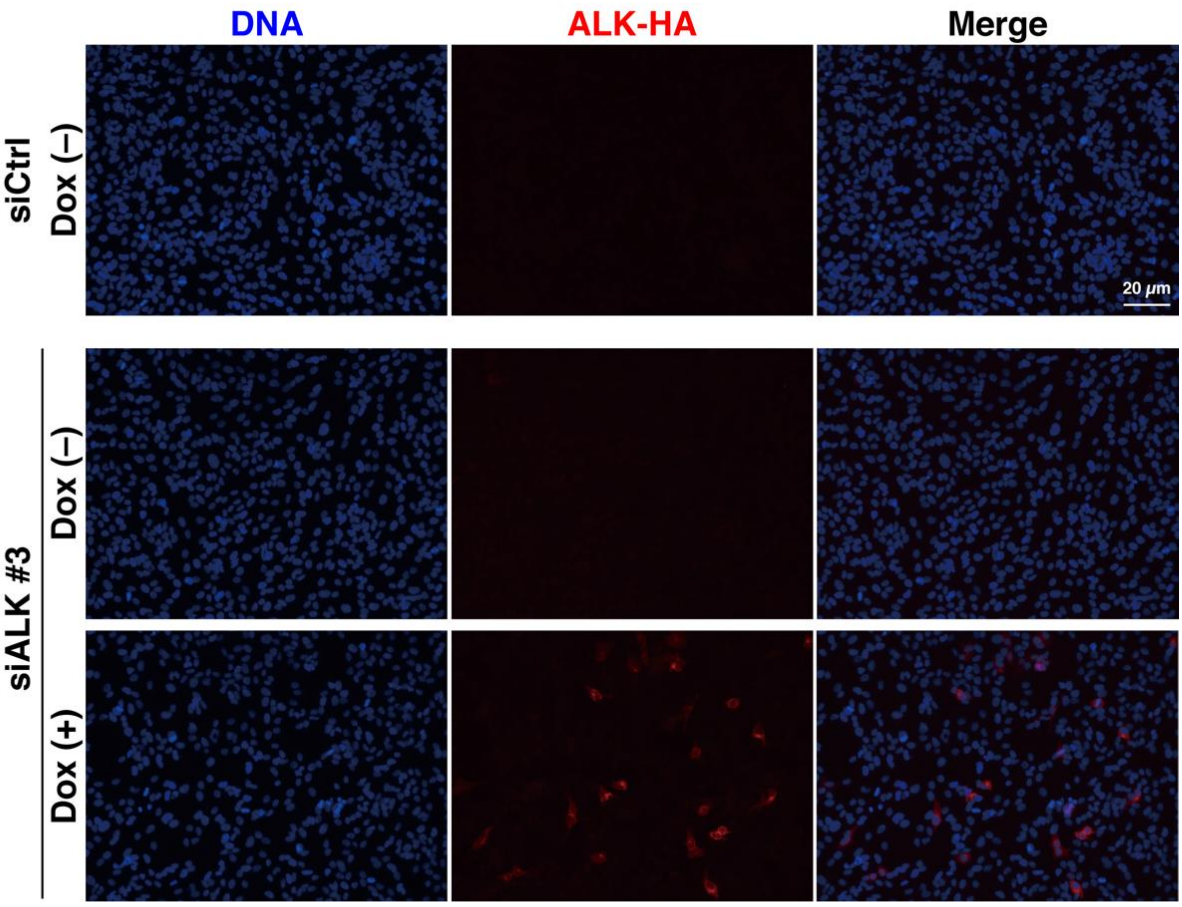

Figure S4. Inducible expression of ALK-HA in Dox-treated cells. SH-SY5Y/ALK-HA cells were transfected with 3′-UTR-targeting siALK (siALK #3) for 72 h with or without Dox. Microscopic images were taken at 10× objective. Scale bar, 20 μm.

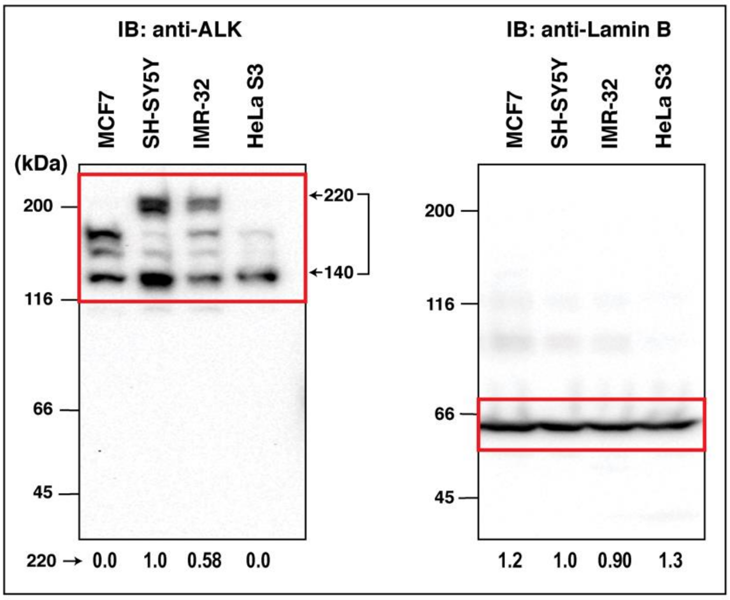

Full-length blots of Figure 1A.

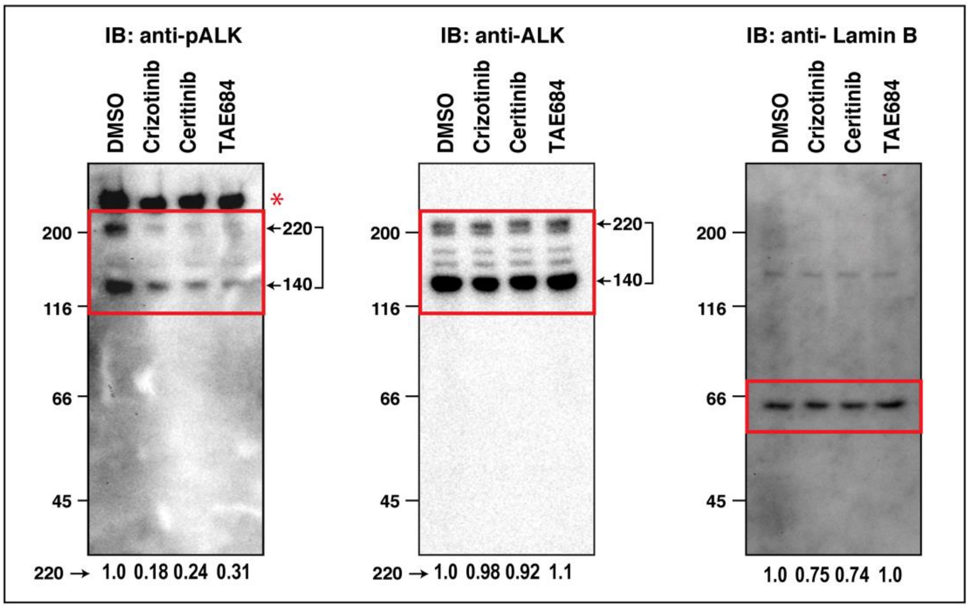

Full-length blots of Figure 1E.

Figure S5. Full-length blots of Figure 1. The band intensities were measured and their ratios to that in SH-SY5Y (upper panel) or DMSO-treated cells (lower panel) are shown below the blots. Asterisk indicates non-specific bands.

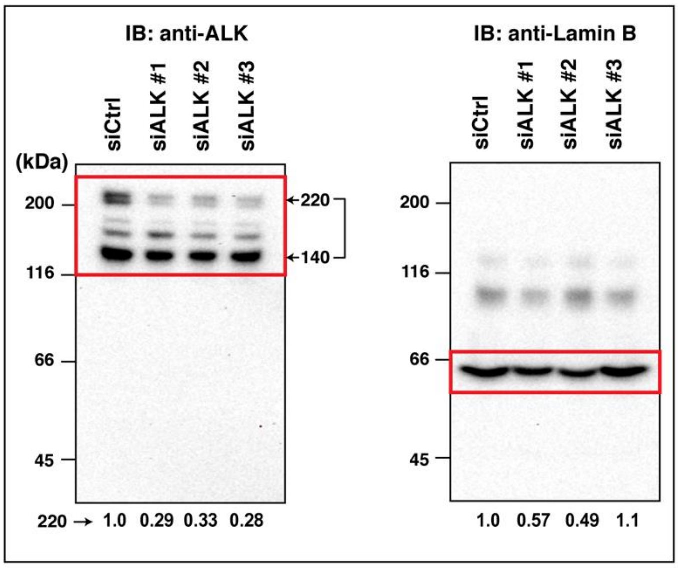

Full-length blots of Figure 3A.

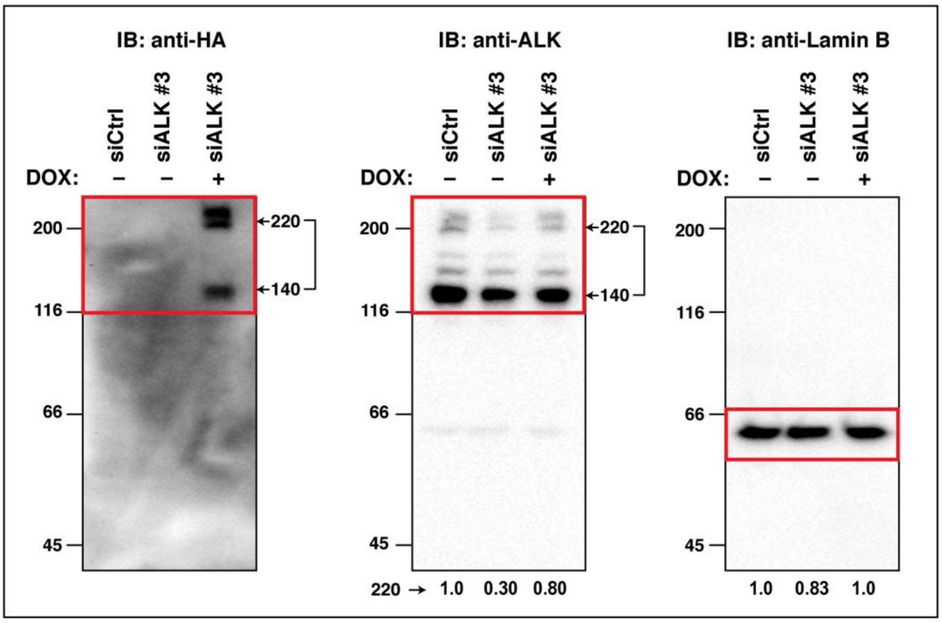

Full-length blots of Figure 3D.

Figure S6. Full-length blots of Figure 3. The band intensities were measured and their ratios to that in siCtrl-treated cells are shown below the blots.

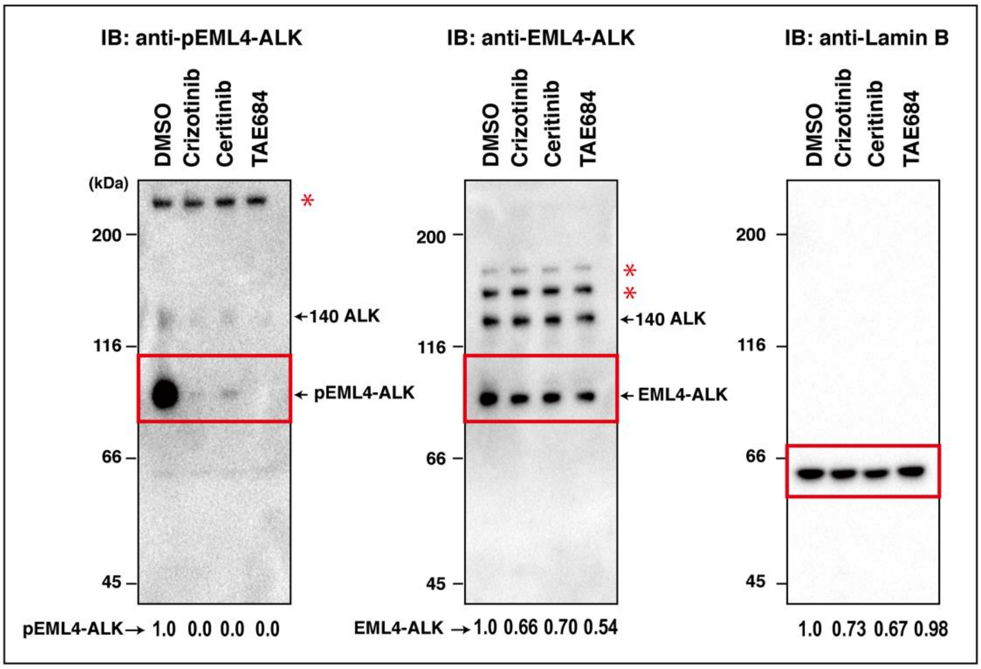

Full-length blots of Figure 6B.

Figure S7. Full-length blots of Figure 6. The band intensities were measured and their ratios to that in DMSO-treated cells are shown below the blots. Asterisks indicate non-specific bands.

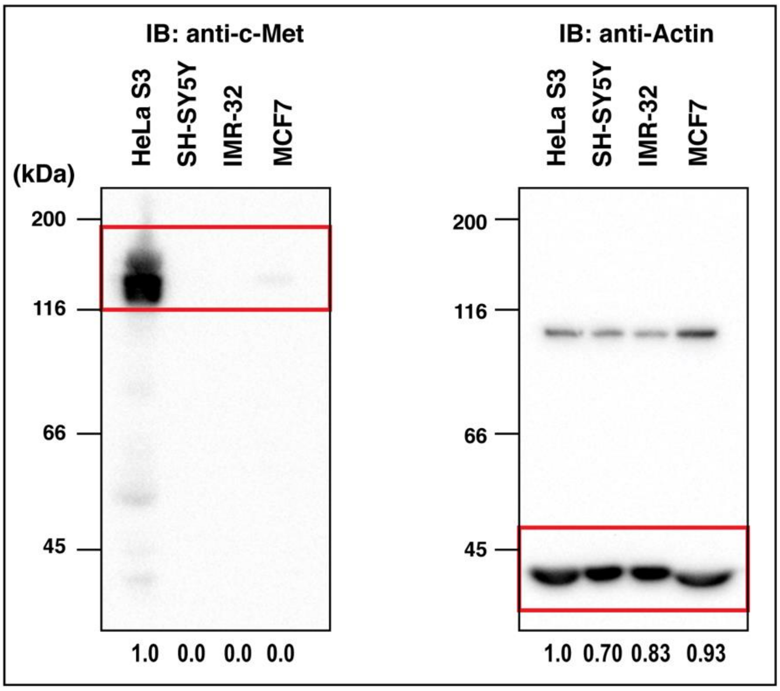

Full-length blots of Supplementary Figure S2A.

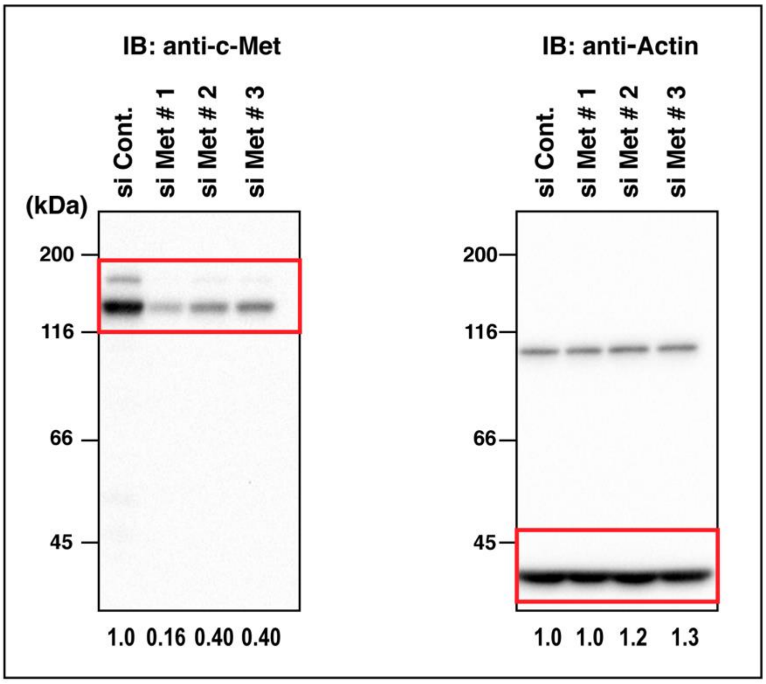

Full-length blots of Supplementary Figure S2D.

Figure S8. Full-length blots of Figure S2. The band intensities were measured and their ratios to that in HeLa S3 (upper panel) or siCtrl-treated cells (lower panel).

© 2020 by the authors. Submitted for possible open access publication under the terms and conditions of the Creative Commons Attribution (CC BY) license (http://creativecommons.org/licenses/by/4.0/).
